# Flea-borne *Bartonella grahamii* and *Bartonella taylorii* in Bank Voles

**DOI:** 10.3201/eid1004.030455

**Published:** 2004-04

**Authors:** Kevin J. Bown, Malcolm Bennett, Michael Begon

**Affiliations:** * University of Liverpool, Liverpool, United Kingdom

**Keywords:** *Bartonella grahamii*, *B. taylorii*, *Ctenophthalmus nobilis*, transmission

## Abstract

*Bartonella* species are increasingly associated with a range of human and animal diseases. Despite this, we have a poor understanding of the ecology and epidemiology of many species, especially those circulating in wild populations. Previous studies have demonstrated that a diverse range of *Bartonella* species are abundant in wild rodent populations; little is known regarding their modes of transmission, although both direct and indirect routes have been suggested. In this study, with bank voles (*Clethrionomys glareolus*) as the host species, we demonstrate that the rodent flea *Ctenophthalmus nobilis* is a competent vector of at least two *Bartonella* species, *B. grahamii*, which has previously been associated with human infection, and *B. taylorii*. In contrast, no evidence of either horizontal or vertical transmission was seen in bank voles inoculated with *B*. *taylorii* maintained in an arthropod-free environment; this finding suggests that fleas may be essential for transmitting some *Bartonella* species.

The genus *Bartonella* currently contains 19 species of gram-negative bacteria that parasitize the erythrocytes of vertebrate hosts, and an increasing number of species are now considered as emerging infections of medical and veterinary importance (*1*). In addition to humans and domesticated animals, they have also been isolated from a variety of wild mammal species, including cervids, ruminants, carnivores, and rodents (*1*–*3*). Of these, rodents are perhaps the best studied, with high prevalences of *Bartonella* infections, coupled with highly diverse species and strains (*4*–*7*). Human infections with *Bartonella* species of rodent origin have been reported from both sides of the Atlantic: in the United States, *B. elizabethae*, associated with endocarditis, *B. washoensis*, associated with cardiac disease, and *B. vinsonii* subsp. *Arupensis*, causing fever and neurologic symptoms (*8*–*10*); in Europe, *B. grahamii,* isolated from the eye of a patient with neuroretinitis (*11*).

While the association of *Bartonella* of rodent origin with human disease continues to increase, our understanding of the ecology and epidemiology of these infections is scant. Fundamental to this endeavor would be clarifying their mode(s) of transmission. *Bartonella* species are generally considered to be transmitted by arthropod vectors, and *Bartonella* DNA has been detected in fleas and ticks collected from both wild and domestic animals (*12*–*16*). However such findings do not necessarily prove vector competence, and vectors have only been conclusively identified for a few species: the sand fly (*Lutzomyia verrucarum*) for *B. bacilliformis* (*17*), the body louse (*Pediculus humanus*) for *B. quintana* (*18*), and the cat flea (*Ctenocephalides felis*) for *B. henselae* (*19*). Anecdotal evidence exists for the role of ticks as vectors of at least some *Bartonellae* (*20*–*22*). For rodent *Bartonellae*, two vectors have been suggested. The oriental rat flea (*Xenopsylla cheopis*) was demonstrated to be a competent vector of an unidentified *Bartonella* species that infected bank voles (*Clethrionomys glareolus*) (*23*), and the vole ear mite (*Trombicula microti*) was proposed as a vector of *B. vinsonii* (*24*). However, no experimental transmission studies have been undertaken in which the *Bartonella* species involved could be accurately identified by, for example, using a molecular approach. In addition, vertical transmission has been suggested as a potential mechanism by which infection may be maintained within a population (*25*), and experimental data suggest that transplacental transmission occurred in BALB/c mice infected with *B. birtlesii*, although no viable fetuses were bacteremic (*26*).

The aim of this study was to determine the potential for fleas, collected from a population of bank voles in which *Bartonella* infections were known to be endemic, to transmit infection to naïve bank voles. In addition, the potential importance of direct horizontal or vertical transmission was investigated.

## Materials and Methods.

Twenty fleas were collected from six bank voles sampled in a mixed woodland in northwest England (53°20.6N, 3°02.4W) where previous studies had shown the prevalence of *Bartonella* infection in bank voles was approximately 60% (*5*). These fleas were added to a rodent “arena,” measuring 1.2 m x 1.2 m in a temperature-controlled room. The arena contained sawdust as substrate, hay and shredded paper as bedding, and Longworth traps (Abingdon, UK) set on prebait as nest boxes. No *Bartonella* spp*.* had been used in experiments in the arena before the introduction of the fleas, and no fleas had previously been kept in the arena. The arena had been kept free of bank voles for 2 weeks before this study began. Twenty-eight captive-bred bank voles from a *Bartonella-*free colony maintained at the University of Liverpool were added to the arena immediately after the fleas were introduced. All of these voles had tested negative for *Bartonella* infection before entering the arena.

Four weeks after the bank voles were added to the arena, all were euthanized, and blood samples were collected by cardiac puncture. In addition to the 28 voles originally introduced to the arena, one female had produced a litter, and the two pups produced were also humanely killed and had sterile blood samples collected. Fleas were collected from all rodents and kept in individual tubes (1 per rodent) containing 70% ethanol. A sample of fleas from bedding within the arena was collected at the same time. All fleas were identified to species level (*27*).

Isolation of *Bartonella* spp. from the blood samples was undertaken by plating freeze-thawed blood onto Colombia blood agar plates enriched with 5% horse blood. Plates were incubated at 37^°^C and 5% CO_2_ for up to 14 days. Isolates putatively resembling *Bartonella* spp. colonies were further characterized by polymerase chain reaction (PCR). Individual colonies were prepared by boiling in 100 μL of sterile deionized distilled water for 10 min. Five microliters of this preparation were used as template. Each 50-μL reaction contained 1.25 U of Taq polymerase, 200 mM of each dNTP, 1.5 mM of MgCl_2_, and 30 pmol of each primer. Initial characterization used primers QHVE1 and QHVE3 (*28*) that target the 16S–23S rRNA intergenic spacer region. PCR products from positive samples were purified by using the Promega Wizard PCR Preps kit (Promega, Madison, WI), and then digested with *Hae*III as previously described (*29*). Samples relating to each REA pattern were then analyzed by using primers BhCS781.p and BhCS1137.n (*30*), which target the citrate synthase (*gltA*) gene. After purification these PCR products were sequenced with an ABI 377 automated sequencer, and the sequences were compared with previously published sequences by using the BLAST program from the National Center for Biotechnology Information Web site (available from: http:/www.ncbi.nlm.nih.gov/BLAST/).

Horizontal and vertical transmission experiments were undertaken using 16 bank voles, housed, in the absence of fleas, in cages containing male-female pairs approximately 4 weeks of age, one or both of which was injected through the footpad with approximately 10^6^ CFU of *Bartonella taylorii*. In two of the cages both voles were inoculated, in two only the male vole was inoculated, and in four others only the female vole was inoculated. Pairs were kept until they had produced a litter. Blood samples were taken at day 0, when the voles were inoculated, at day 10 to confirm infection status of the adults, and 8 weeks later when litters were between 7 and 14 days old. Isolation attempts were carried out as described.

## Results

Twenty-one of the 28 blood samples from the bank voles produced colonies resembling *Bartonella* spp., and all of these were confirmed as *Bartonella* spp. by PCR. Restriction enzyme analysis of the resulting PCR products showed that two different *Bartonella* genotypes were present in the bank voles (Figure). Sequence analysis of the *gltA* gene showed these to represent *B. taylorii* (16 isolates) and *B. grahamii* (6 isolates) (one bank vole was coinfected with both). In addition to the original 28 voles added to the arena, two pups were sampled that had been suckling from a bacteremic female. Neither was bacteremic.

**Figure:**
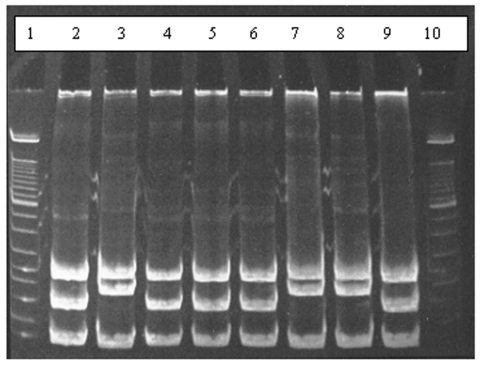
Results of restriction enzyme analysis on 7% polyacrylamide gel showing the two restriction patterns obtained by digesting polymerase chain reaction products with *Hae*III. Lanes 2, 4, 5, 6, and 9 show digestion of amplicons of *Bartonella* *taylorii*; lanes 3, 7, and 8 show digestion of *B. grahamii* amplicons; lanes 1 and 10 contain molecular weight markers.

A total of 217 fleas were collected from the 28 bank voles (mean 7.75 fleas per vole). Only one species of flea was identified, *Ctenophthalmus nobilis nobilis*. Ten pools of five randomly selected fleas collected from the voles were tested for *Bartonella* spp. DNA using the *gltA* PCR. All pools tested positive, and of 10 individual fleas collected directly from the arena itself, 7 tested positive for *Bartonella* spp. DNA. Four were positive for *B. taylorii*, one for *B. grahamii*, and two for both.

None of the naïve adults involved in the horizontal transmission experiment acquired infection directly from its mate, despite that all inoculated animals remained bacteremic throughout the experiment. Seven of the eight pairs of voles produced a litter, one of the pairs in which the female alone was inoculated did not. A total of 20 young were produced from the seven litters, with litter sizes ranging between one and five offspring (mean 2.86 offspring per litter). No bacteremia could be detected in any of the offspring, whether only one or both parents had been inoculated.

## Discussion

This study shows that fleas are efficient vectors of at least some rodent bartonellae. Twenty one of 28 (75%) naïve bank voles housed with wild-caught fleas for 4 weeks became bacteremic, 16 voles (57.1%) infected with *B. taylorii* and 6 voles (21.4%) infected with *B. grahamii.* Similarly, each of 10 pools of 5 fleas collected showed the presence of *Bartonella* spp. DNA within them, and 7 of 10 individual fleas were also positive. Fleas have previously been implicated in the transmission of *B. henselae* infections of cats (*19*,*31*,*32*), and *Bartonella* DNA has previously been detected in fleas collected from rodents (*14*,*15*), but no recent experimental studies on the role of fleas in the transmission of rodent bartonellae have been reported since early studies by Krampitz (*23*) indicated that fleas could transmit an unidentified *Bartonella* species. In fact, two different *Bartonella* species could be transmitted by a single species of flea, suggesting little vector-bacteria specificity.

On the other hand, no transmission occurred between infected and susceptible animals when housed together in the same cage in the absence of fleas, and no transmission could be detected from parent to offspring, although larger numbers of animals may be needed to confirm that such transmission does not occur. This absence of vertical transmission agrees with results of a study of cats infected with *B. henselae* (*33*,*34*), but Kosoy and colleagues (*25*) found that *Bartonella* could be isolated from the neonates and embryos of naturally infected North American rodents, while transplacental transmission of *B. birtlesii* was also reported in BALB/c mice, although none of the viable offspring in that study were bacteremic (*26*).

These findings are of potential public health importance: *B*. *grahamii* has previously been associated with human disease (*11*), although the pathogenic potential of *B. taylorii* is as yet unknown. Furthermore, the implication of fleas in the transmission of these rodent *Bartonella*, as well as *B. henselae* (*19*,*31*) suggests that fleas may be involved in the transmission of many other rodent *Bartonella* species, some of which have already been shown to be pathogenic to humans. Whether the route of rodent to human transmission is likely to be due to flea transmission or through direct contact, such as bites or scratches as is commonly the case for *B. henselae* (*1*), needs to be investigated.

The exact route by which fleas transmitted *Bartonella* to susceptible rodents remains unclear. Future work should seek to distinguish the role of fecal contamination and then the role of scratching (*32*) from direct transmission through feeding. Investigating the efficiency of different flea species in transmitting a variety of *Bartonella* species would be valuable as would determining whether fleas infected with a number of *Bartonella* species transmit one species more efficiently than the others. Studies such as these would help expand the current knowledge on vector-*Bartonella* specificity and determine its importance in influencing the diversity of *Bartonella* species.
